# The Vector - Host - Pathogen Interface: The Next Frontier in the Battle Against Mosquito-Borne Viral Diseases?

**DOI:** 10.3389/fcimb.2020.564518

**Published:** 2020-10-16

**Authors:** Maria Gorreti Onyango, Alexander T. Ciota, Laura D. Kramer

**Affiliations:** ^1^New York State Department of Health, Wadsworth Center, Slingerlands, NY, United States; ^2^School of Public Health, State University of New York at Albany, Albany, NY, United States

**Keywords:** mosquito-borne viruses, mosquito, vector-host-pathogen, saliva, microbiome

## Abstract

An unprecedented spread of mosquito-borne viruses and increasing populations of mosquito vectors has led to an increase in the frequency of mosquito-borne virus disease outbreaks. Recent outbreaks of Zika virus (ZIKV) and yellow fever virus (YFV), among others have led to a concerted effort to understand the biology of mosquito-borne viruses and their interaction with their vector mosquito and vertebrate hosts. Recent studies have aimed to understand the vector-host-pathogen interface and how it influences infection, tropism and disease severity in the vertebrate host. The initial replication of the pathogen at the skin bite site is crucial in determining the progression of the infection in the vertebrate host. Delineating the role of the commensal microbes in the mosquito saliva as well as how they interact with the vertebrate host keratinocytes will improve our understanding of disease immunopathology and may lead to new therapeutics.

## Introduction

Mosquito-borne viruses consist of a group of pathogens including dengue virus (DENV), Zika virus (ZIKV), chikungunya virus (CHIKV) and West Nile virus (WNV) which co-circulate geographically and result in significant global health challenges (Weaver and Reisen, 2010). Population growth, deforestation, urbanization, climate change and movement of people, animals and vectors have led to recent increases in the frequency and distributions of disease outbreaks associated with mosquito-borne viruses (Roiz et al., 2014; Vasilakis and Gubler, 2016). Mosquito-borne viruses affect millions of people globally. In spite of this, several of the diseases resulting from infection with these viruses have no clinically approved therapy or vaccine to prevent them and medications for symptomatic relief are the only source of comfort for infected individuals (Jackman et al., 2019; Kazmi et al., 2020). Control measures based on avoiding mosquito bites and controlling the mosquito vector are the main prevention and control measures against most mosquito-borne viruses (Petersen et al., 2016).

At the bite site, mosquito saliva elicits an immune response in the vertebrate host, enhancing dissemination of the pathogen (Mellink and Vos, 1977; Titus et al., 2006; Styer et al., 2011; Cox et al., 2012; Conway et al., 2014; Pingen et al., 2017). Specific saliva components have been studied for their modulatory effect on blood feeding (Kerlin and Hughes, 1992; Ribeiro, 1992; Arca et al., 1999; Arcà et al., 2007) as well as on pathogen transmission in vertebrate hosts (Conway et al., 2014, 2016; Jin et al., 2018; Uraki et al., 2019). No study has described the role of mosquito salivary microbes as potentiating factors of virus pathogenicity, yet studies suggest that factors other than salivary proteins may contribute to disease-enhancing features of pathogen -transmission by vector bites (Dey et al., 2018).

## Vector Pathogen Host Interface and its Influence on Arbovirus Transmission

The successful transmission of a vector-borne pathogen depends on unique interactions within a triad of interactions between pathogen -host and vector (Manning and Cantaert, 2019).

Mosquito infections with pathogens prompts complex crosstalk between different metabolic and immune pathways which include the immune deficiency (lmd), the Toll and Janus kinases and signal transducers and activators of transcription (JAK-STAT). RNA interference (RNAi) modulates virus replication (Hegde et al., 2015; Bartholomay and Michel, 2018). Autophagy, apoptosis and oxidative stress can trigger and alter the immune pathways of the arthropod (Champion and Xu, 2017). On the other hand, the alterations of host metabolic pathways by transmitted pathogens is through the modulation of stress-inducible genes involved for instance in redox and detoxifying enzyme metabolisms (Guégan et al., 2018). The holobiont concept has demonstrated that symbiotic bacteria play an important role in host biology (Guégan et al., 2018)^*^.

*Wolbachia*, an endosymbiotic bacterium naturally found in 40% of insects has been studied extensively for its anti-viral activity. Indeed, *Wolbachia* has been shown to confer an anti-viral effect by modifying the host intracellular environment. A competitive utilization of amino acids between the host and *Wolbachia* has been demonstrated (Caragata et al., 2014). This results in an altered host environment less optimal for incoming virus but favoring *Wolbachia*'s reproduction and transmission (Lindsey et al., 2018).

Further, presence of *Wolbachia* induces cellular stress by increased expression of the host antioxidant proteins superoxide dismutase, peroxiredoxin and glutathione peroxidase, as well as the *Wolbachia* proteins superoxide dismutase and bacterioferritin (Brennan et al., 2008). The enrichment of reactive oxygen species (ROS) could potentially lead to disruption of biological macromolecules such as proteins, nucleic acids and lipids at physiological levels and activation of signaling pathways such as the extracellular signal-regulated kinase (ERK) pathway known to be critically important in mediating protection against RNA viruses such as Sindbis virus (SINV) (Müller et al., 1997; Milligan et al., 1998; Victor and Barry, 2000; Xu et al., 2013).

In spite of the immune reaction mounted, arboviruses are generally known to establish a lifelong infection in vectors (Althouse and Hanley, 2015). Arthropod-mediated transmission of arbovirus is preceded by a blood meal uptake by a mosquito resulting in the mosquito injecting the virus into the human skin (Briant et al., 2014).

Mosquito are known to possess salivary factors directly modulating host immune defenses that peak after completion of engorgement (Schneider and Higgs, 2008)^*^. This is important because the modification of the local environment at the feeding site which is the point of introduction of a pathogen by the mosquito where resident and migratory cells encounter the pathogen (Pingen et al., 2016; Arcà et al., 2017).

Skin cells such as epidermal keratinocytes (Surasombatpattana et al., 2011), dendritic cells (Lozach et al., 2005) or neurons (Salazar et al., 2013) are the initial targets of arboviruses upon introduction at the skin bite site. The virus attaches to the cell surface and by receptor-mediated endocytosis, gains entry into the cell, resulting in the release of the nucleocapsid and the viral RNA into the cytoplasm (Rey et al., 1995). The replication of the virus happens in the rough endoplasmic reticulum and golgi-derived membranes (Salonen et al., 2004; Mackenzie, 2005). Subsequently, the virus is packaged into progeny virion or alternatively utilized to translate additional proteins (Rey et al., 1995).

Sensing a viral infection, innate immune system of the vertebrate host acts as the first line of defense. The initial response involves the recognition of the pathogen-associated molecular patterns (PAMP) expressed by the virus in non-immune cells or cells of the innate immune system such as monocytes or macrophages, dendritic cells and natural killer cells by pattern recognition receptors (PRRs), such as the Toll-like receptor (TLR)-family or RIG-I like receptor (Ye et al., 2013; Hamel et al., 2016). After the detection of PAMPs, several signaling pathways are triggered resulting in direct secretion of interferons (IFNs) as well as expression of numerous Interferon-Stimulated Genes (ISGs) generating an antiviral state (Hamel et al., 2016).

In spite of these robust immune responses at the early onset of invasion, viruses have developed means of evading the host immune system response. Within hours of infection, many pathogens induce specialized cellular proteins also known as pathogen-recognition receptors (PRRs) which include Toll-like receptors 3 and 7(TLR3 and TLR7) (Wang et al., 2004, 2006; Daffis et al., 2008a,b; Tsai et al., 2009), retinoic acid-inducible gene 1 (RIG-I) and melanoma differentiation associated gene 5 (MDA5) (Fredericksen et al., 2004, 2008; Chang et al., 2006; Fredericksen and Gale, 2006; Kato et al., 2006). Through a mechanism yet unknown, virulent WNV strains evade PRRs detection, giving them an upper hand to replicate within the cells during the early stages of infection (Fredericksen and Gale, 2006).

Establishment of the systemic viral infection depends on different factors including the vertebrate host response to mosquito saliva (Styer et al., 2011; Cox et al., 2012; Conway et al., 2014; Pingen et al., 2017). Mosquito feeding enhances the disease severity in the vertebrate host (Edwards et al., 1998; Peters et al., 2008; Styer et al., 2011; Cox et al., 2012). Indeed, the bite of a mosquito results in an influx of neutrophils and the proliferation of myeloid cells that are permissive to infection by viral pathogens (Cox et al., 2012; Pham et al., 2012; Pingen et al., 2016). Furthermore, the mosquito bite site contains extensive edema that allows virus inoculum retention at the bite site facilitating the infection of the cutaneous cells (Pingen et al., 2016).

## Mechanisms of Mosquito Saliva Enhancement of Arbovirus Infection

The underlying molecular mechanisms by which the mosquito saliva modulates the vertebrate immune system are still not fully understood, but it is known that salivary factors can modulate the functions of human cells and thus enhance or suppress viral replication and interfere with the establishment and systematic replication of the virus (Wichit et al., 2016) (Table 1).

**Table 1:** Mosquito saliva proteins associated with modulation of arbovirus transmission.

**Saliva protein**	**Source**	**Virus**	**Model of study**	**References**
AgBR1	*Ae. aegypti*	ZIKV	Ifnαr^−/−^ Ifn⋎r^−/−^ mice (AG129-SV129 background)	Uraki et al., 2019^*^
D7	*Cx. tarsalis*	WNV	C57/BL6 strain mice	Reagan et al., 2012^*^
Serine protease CLIPA 3	*Ae. aegypti*	DENV	IFNAR^−/−^ mice	Conway et al., 2014^*^
AaSG34	*Ae. aegypti*	DENV-2	Stat1^−/−^ mice	Sri-in et al., 2019^*^
LTRIN	*Ae. aegypti*	ZIKV	I*fnαr*^−/−^ and L*tbr*^−/−^ mice	Jin et al., 2018^*^
Adenosine deaminase	*Ae. aegypti*	DENV	Human keratinocytes	Surasombatpattana et al., 2014^*^
Aegyptin	*Ae. aegypti*	DENV	IRF-3/7^−/−−/−^ mice	McCracken et al., 2014^*^
AaVA-1	*Ae. aegypti*	DENV; ZIKV	AG6 mice (ifnar1^−/−^ifngr1^−/−^)	Sun et al., 2020^*^
NeSt1	*Ae. aegypti*	ZIKV	*Ifnαr*1^−/−^*Ifnγr*^−/−^ mice (AG129–SV129 background)	Hastings et al., 2019^*^

* *References of significant relevance*.

A 15 kDa protein, LTRIN identified in *Ae. aegypti* salivary glands, enhanced the transmission of ZIKV in *Ifn*α⋎ ^−/−^ mice (but not wild type mice) by binding to lymphotoxin-β receptor (LTβR) thus inhibiting signaling via the transcription factor NF-_K_B and subsequent production of inflammatory cytokines (Jin et al., 2018). Lymphotoxin is a member of the tumor necrosis factor (TNF) family of cytokines responsible for the regulation of the growth and function of lymphocytes. The LTβR signaling pathway facilitates crosstalk between epithelial cells (the first line of defense against mucosal pathogens) and the immune cells and LTβR-deficient mice have been shown to be more susceptible to various pathogenic infections (Ware, 2005; Wang et al., 2010).

Indeed, a 34 kDa *Ae. aegypti* saliva protein completely inhibited type I IFN expression by significantly suppressing the expression of both IFN – regulatory factors (IRF 3 and 7). The protein exerted a dose-dependent effect against the expression of AMP, hence enhancing the replication of DENV in the human keratinocytes by blocking the IRF signaling pathway (Surasombatpattana et al., 2014)^*^. Furthermore, *Ae. aegypti* D7 saliva protein was demonstrated to inhibit DENV infection in murine models by a direct interaction with DENV envelope protein (Conway et al., 2016).

Mosquito saliva enhanced CHIKV pathogenesis in a murine study by significantly downregulating inflammatory genes like TLR-3, IL-2, IFN-⋎,TNF-α and IFN-β, while enhancing anti-inflammatory genes like IL-4 and IL-10 as well as induced cellular infiltration of transmigrated inflammatory cells in dermal parenchyma (Agarwal et al., 2016). Further, it was observed that CHIKV-infected human fibroblasts in the presence of *Ae. aegypti* saliva demonstrated marked decrease of type I IFN-responsive genes as well as a downregulation of phosphorylated form of STAT2, hence showing that mosquito saliva affects the host immune response via the JAK-STAT signaling pathway leading to diminished immune response and enhanced CHIKV replication (Wichit et al., 2016).

Infection of cultured macrophages, bone marrow derived dendritic cells as well as mice with WNV in the presence of *Ae. aegypti* saliva resulted in an increase in WNV infection by a reduction in IFN-β levels in microphages and inducible nitric oxide synthase (iNOS) while increasing levels of IL-10. On the other hand, WNV infection was enhanced *in vivo* by a significant reduction of T cells recruitment as well as elevated levels of IL-10 in mice skin and lymph nodes (Schneider et al., 2010).

Apart from the mosquito saliva proteins that enhance viral infection at the bite site, exogenous microRNAs (miRNAs) have been identified in mosquito saliva (Maharaj et al., 2015)^*^. miRNAs are short, 18–24 nucleotide, non-coding RNAs which regulate gene expressions post-transcriptionally by binding complementarily regions, mainly in the 3' UTRs of targeted messenger RNAs. Next -generation sequencing identified several novel *Ae aegypti* and *Ae. albopictus* miRNAs specific to CHIKV-infected mosquitoes and significant inhibition of CHIKV in AAG-2 and BHK-21 cells by miR-184 indicated the importance of this miRNA in alphavirus infection and replication in host cells (Maharaj et al., 2015). Further characterization of the mechanistic basis for these interactions could result in novel therapeutic tools for arboviral control.

In addition, extracellular vesicles such as exosomes have recently emerged as a trojan horse transporting viral particles and genomes to new susceptible hosts (Altan-Bonnet, 2016). Exosomes can carry cellular proteins, lipids, nucleic acids, cytokines, chemokines, translation regulatory factors, toxins and miRNAs between cells (Tkach and Théry, 2016). Indeed, DENV infected *Ae. albopictus* and *Ae. aegypti* cells were shown to secrete extracellular vesicles that contained infectious viral RNA and proteins that were infectious to naïve mosquito and mammalian cells, including human-skin keratinocytes and blood endothelial cells. Further, mosquito extracellular vessicles from ZIKV-infected C6/36 cells infected and activated naïve mosquito and mammalian cells. In addition, C6/36 extracellular vessicles promoted the differentiation of naïve monocytes while inducing a pro-inflammatory state with tumor necrosis factor-alpha (TNF-α) mRNA expression. Lastly, ZIKV C6/36 extracellular vessicles resulted in endothelial vascular damage and increased endothelial permeability (Martínez-Rojas et al., 2020)^*^. Both studies suggest that mosquito exosomes may potentially be excreted in mosquito saliva during blood feeding which may fuse with keratinocytes and endothelial cells and enhance viral transmission from vector to the vertebrate host (Sultana and Neelakanta, 2019).

## Microbiome at the Vector-Host Interface and its Influence on Disease Outcome

Despite the knowledge of the immune modulatory role of a number of salivary proteins at the vector- pathogen- vertebrate interface (Reagan et al., 2012; Conway et al., 2014; McCracken et al., 2014; Surasombatpattana et al., 2014; Jin et al., 2018; Hastings et al., 2019; Sri-in et al., 2019; Uraki et al., 2019; Sun et al., 2020), a gap exists in knowledge of the impact of the interactions between salivary associated bacterial flora and the vertebrate host (Sharma et al., 2014; Finney et al., 2015).

*Asaia*, a bacterium that dominates mosquito-associated microbiota, has been identified in the saliva of *Anopheles stephensi* and *A. gambiae* (Favia et al., 2007; Damiani et al., 2010). Fluorescent-labeled *Asaia* successfully colonized 32% of salivary glands of colony-reared *Anopheles stephensi* mosquitoes demonstrating that gut microbiota can efficiently cross tissue barriers and colonize peripheral organs (Favia et al., 2007)^*^.

Further, (Sharma et al., 2014)^*^, applied a metagenomics approach to study the mosquito salivary gland microbial community structure of laboratory reared *A. culicifacies* demonstrating that mosquito salivary glands contain more complex microbial communities than the midgut, dominated by bacteria of the phylum Proteobacteria. The results showed that bacteria are equally associated with each of the three salivary lobes, very important tissues associated with pathogen transmission. The potential for interactions between co-existing arboviruses and microbes in the salivary gland and saliva of mosquitoes to influence transmissibility is completely unstudied. Upon skin penetration, the mosquitoes move their stylets back and forth to locate a blood vessel. During this probing phase, mosquitoes salivate copiously (Griffiths and Gordon, 1952^*^; Ribeiro, 1984). The possibility of introduction of bacterial microbes to the bite site via the mosquito saliva is plausible.

These microbes could interact with the skin epidermis and dermis at the point of introduction during blood meal intake. Understanding the role of salivary microbes on virus transmission and virus tropism in the vertebrate host could reveal novel targets for the control of mosquito-borne diseases.

## Discussion

Global changes resulting in increases in density and geographic expansion of mosquito populations is a public health threat likely to result in emergence or re-emergence of mosquito-borne diseases globally. Innovative, comprehensive and cohesive research efforts focusing on interactions with the vector and the human host are needed.

Great strides have been made in the understanding of the interactions between the arboviruses, the vector and the vertebrate hosts. However, there has been an under exploration of the knowledge of the functions of mosquito-associated bacteria. Indeed, the development of techniques such as metagenomics, metatranscriptomics, metaproteomics, and metabolomics creates opportunities to improve the mechanistic knowledge of the molecular interactions of the mosquito holobiome.

Genetic engineering of mosquitoes to enhance vector immune or antiviral responses or manipulation of the vector microbiome to reduce vector competence or vector populations are methods of mitigation currently being explored to reduce the incidence of arboviruses. The development of new technologies such as CRISPR is beneficial for enabling gene manipulation of mosquitoes as well as enabling gene drive that could result in modified mosquito populations with decreased capacity for pathogen transmission (Huang et al., 2019).

While strategies focusing on population reduction and competence are promising, targeting the vector-host interface could lead to the discovery of novel control and therapeutic strategies.

Previous studies demonstrate a clear role for components of mosquito saliva influencing transmission and disease, yet the relevance of saliva bacterial microbes in the vector- host interactions is still largely unknown.

The identification of metabolically active bacteria and the discovery of novel bacterial genes modulating chemical and biological processes during viral transmission could lead to development of novel transmission barrier tools. Studies that experimentally manipulate saliva microbial profiles could further evaluate the effect of co-transmission on host disease outcome. Together, such studies could offer unique insights into the role of holobiome in vector-host interactions.

## Author Contributions

MO: drafted the paper. AC: participated in the writing of the paper. LK: participated in the writing of the paper. All authors contributed to the article and approved the submitted version.

## Conflict of Interest

The authors declare that the research was conducted in the absence of any commercial or financial relationships that could be construed as a potential conflict of interest.
